# Water vapor sorption behavior of bamboo pertaining to its hierarchical structure

**DOI:** 10.1038/s41598-021-92103-4

**Published:** 2021-06-16

**Authors:** Qi Chen, Changhua Fang, Ge Wang, Xinxin Ma, Junji Luo, Meiling Chen, Chunping Dai, Benhua Fei

**Affiliations:** 1 Department of Biomaterials, International Centre for Bamboo and Rattan, No. 8, Futong East Street, Chaoyang District, Beijing, People’s Republic of China; 2 SFA and Beijing Co-built Key Laboratory of Bamboo and Rattan Science and Technology, State Forestry Administration, Beijing, People’s Republic of China; 3 Department of Wood Science, Faculty of Forestry, University of British Columbia, Vancouver, V6T1Z4 Canada

**Keywords:** Biomaterials, Fluid dynamics, Materials science

## Abstract

Bamboo is an anisotropic, hierarchical, and hygroscopic material. Moisture transport in bamboo is one of the most fundamental properties affecting almost all other physical and mechanical properties of the material. This study investigated the water vapor sorption behaviors of bamboo at various structural levels: cell walls, cells (with pits) and bamboo blocks. The specimens with two sorption directions, longitudinal (L) and transverse (T), were measured by saturated salt solution method and dynamic vapor sorption. The parallel exponential kinetics model was used to analyze the sorption kinetics. The results showed that at the cell wall level, the sorption rate and equilibrium moisture content (EMC) of cell wall in the L specimens were larger than those in the T specimens. The differences were probably caused by the looser cell wall layers in the L specimens. At the cellular scale, pits in the cell wall resulted in an enhanced sorption rate and EMC of the T specimens compared with the L specimens where the pits in the parenchyma cells were only distributed in the lateral walls but not in end walls. At the macro scale, the sorption rate and moisture content of bamboo blocks were largely controlled by the vessel cells. As a hierarchically-structured plant, bamboo performs the biological function of moisture transport at all these scales. This work helps improve the understanding of water transport behavior in bamboo, which may lead to better bamboo drying and impregnation processes.

## Introduction

Bamboo, with the rapid growth rate and high strength, has been extensively used in architecture, bridges, furniture, pulp and paper, bamboo pipes among others^1–5^. Bamboo is a hygroscopic material and its mechanical properties, dimensional stability, and mold resistance are all affected by moisture content^6–9^. As in wood, water in bamboo exists in both forms of free water in the cell lumens and bound water in the cell walls. An accurate knowledge of water transport in bamboo is important to its drying process and chemical treatment. Of particular interest, water transport in bamboo is a multidimensional phenomenon due to its complex structure.

Bamboo has an anisotropic, gradient, and hierarchical structure^10–15^. As an anisotropic material, bamboo has variable water vapor diffusivities which could be analyzed in a cylindrical coordinate system. According to Huang et al.^16^, the water vapor diffusion resistance was markedly lower in the longitudinal direction than in the radial and tangential direction. Chen et al.^17^ measured the equilibrium moisture content (EMC) of bamboo at various radial positions across the culm and found that the EMC of the inner part was higher than that of the outer part especially at high relative humidity (RH) levels, and the outer part provided more sorption sites for water but fewer water clusters than the inner part. Besides the fore-mentioned studies on the anisotropic and gradient behavior, the literature to-date has provided little knowledge about the systematic effect of hierarchical structure on moisture sorption behavior.

In this study, we used the saturated salt solution method and dynamic vapor sorption (DVS) to measure the time-dependent mass response of cell wall substances, cells and blocks of bamboo. Two sorption directions were measured at these scales, and the parallel exponential kinetics (PEK) model was used to describe the sorption kinetics behavior. Emil et al.^18^ mentioned that the PEK model was highly dependent on the specific RH step and hold time. Here, by maintaining the same RH step and the hold time, PEK model was used to compare the scale and directional effects of bamboo. While improving the understanding of the micro and macro sorption mechanisms, this study was carried out with the following objectives:

- To investigate the bound-water sorption behavior of cell walls of bamboo in both longitudinal and transverse directions;
- To evaluate the effect of pits on water vapor sorption; and
- To investigate the water vapor sorption behavior of bulk bamboo in both longitudinal and transverse directions.

## Materials and methods

### Materials

Moso bamboo (*Phyllostachys edulis*), with an age of 5 years, was obtained from Zhejiang Province, China. We confirm that we have the permissions to obtain the Moso bamboo from Zhejiang Province, China. Besides, all local, national or international guidelines and legislation were adhered to in the production of this study. Bamboo culm was selected from the internode sections located at a height between 1.5 and 2.5 m, and was air-dried to constant weight before testing. Bamboo blocks and bamboo slices were prepared for tests.

**Figure 1:**
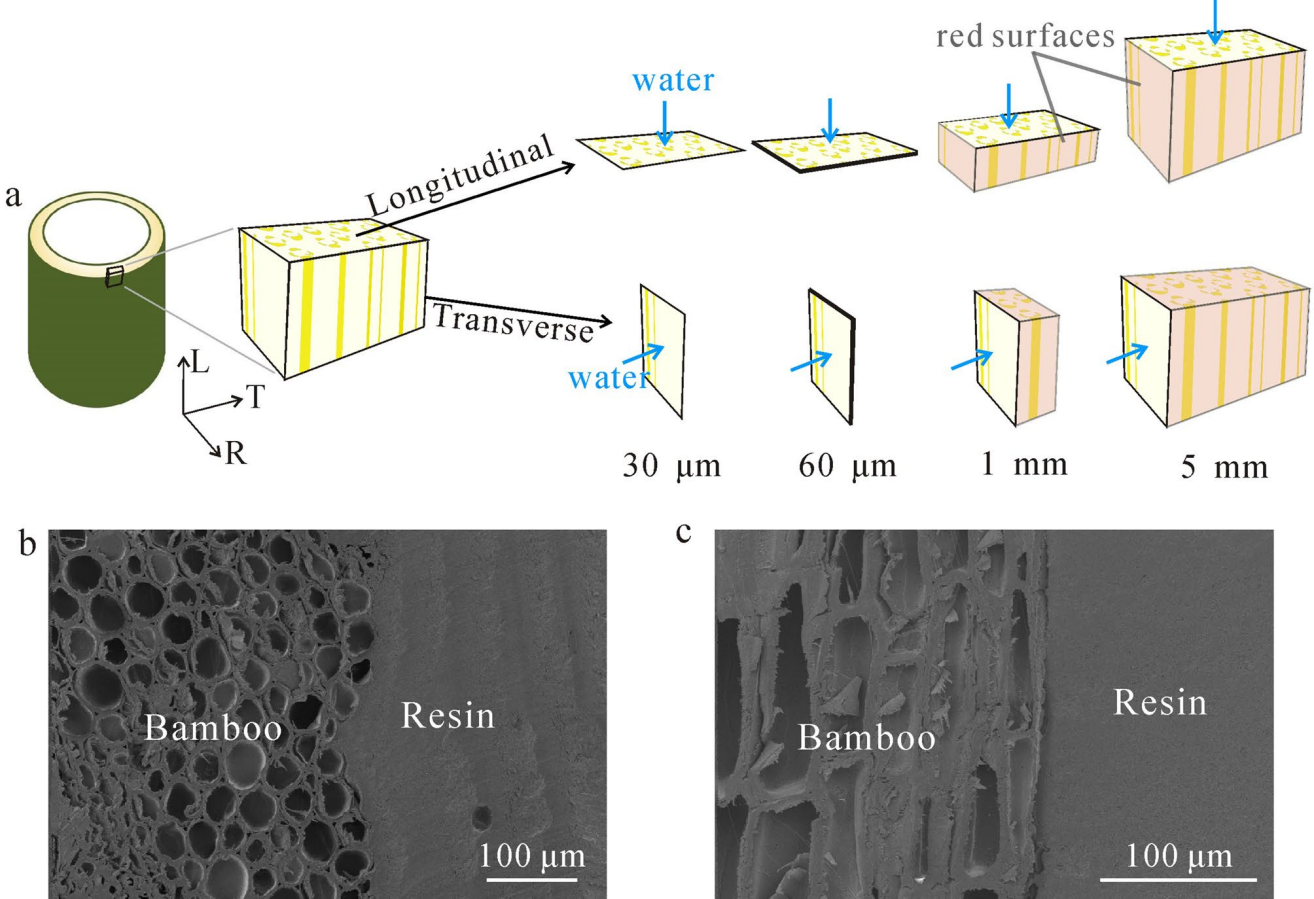
(**a**) Schematic of specimens with longitudinal and transverse sorption direction, where the blue arrows represent moisture sorption directions and red surfaces represent the surfaces that were covered by epoxy resin. (**b**) SEM picture of L specimen with resin edge sealing, (**c**) SEM picture of T specimen with resin edge sealing.

Bamboo blocks with two different sorption surfaces were cut from the stalks (inner and outer portion were removed). When the sorption direction was longitudinal (Fig. 1a), the dimension of specimens was 5 × 2 × L mm (tangential (T) × radial (R) × longitudinal (L)), where L was the sorption thickness (1 and 5 mm). TL and RL surfaces were covered by epoxy resin. When the sorption direction was transverse (Fig. 1a), the dimension of specimens was T × 2 × 5 mm (T × R × L), where T was the sorption thickness (1 and 5 mm). RT and TL surfaces were covered by epoxy resin (Fig. 1a). Figure 1b,c show the L and T specimens with the edges sealed tightly with resin coating, respectively. Very little resin penetrated into the samples.

Bamboo slices (Fig. 1a) with two sorption directions were cut from blocks using a microtome. The thicknesses of the slices were 30 and 60 μm. Without epoxy coating, the water vapor diffusion was assumed to occur mainly through the sorption surfaces because the width and length of slices were 2 and 5 mm, respectively, which were far greater than the thickness.

### Saturated salt solution method

Five specimens were prepared for each thickness. The specimens (bamboo blocks and slices) were placed in the oven at 102 ℃ for drying till they reached a constant weight, and were then placed into desiccators with 90% RH (BaCl_2_ salt solution). Mass of the specimens was measured after 4, 24, 48, 72, 96, 120, 144, 168, and 216 h until equilibrium. For 1 mm and 5 mm samples, the MC was:

$$ {\text{MC}} = \frac{{{\text{W}}_{{\text{t}}}  - {\text{W}}_{0} }}{{{\text{W}}_{{\text{B}}} }} $$

where ${\text{W}}_{\text{t}}$ is the weight of the sample (bamboo and resin) at time t, and ${\text{W}}_{\text{0}}$ is the weight of the sample at the beginning, and ${\text{W}}_{\text{B}}$ is the weight of the dry bamboo.

### Dynamic vapor sorption apparatus

The bamboo slices were tested using a dynamic vapor sorption apparatus (DVS Intrinsic, Surface Measurement Systems Ltd., UK). The sample mass of each set was approximately 35–40 mg, and the sorption processes were run at a constant temperature of 25 °C. The RH was preset to increase from 0 to 90% with an increment of 10% and to 95% with an increment of 5%, and then decreased from 95 to 90% with a decrement of 5% and to 0% with a decrement of 10%. At each stage, samples were kept at the constant RH until the weight change per minute (dm/dt) was less than 0.01% in 10 min, and the mass measurement accuracy of samples was 0.0001 mg. According to previous studies^19^, the DVS technique yields highly reproducible data; therefore, only one measurement for each set was performed in this study.

### Kinetic analysis using PEK model

In the PEK model, water vapor sorption behavior consists of a fast and a slow process^19–22^. The experimental moisture content data obtained by saturated salt solution method was analyzed using the PEK model (Eq. 1) by the Matlab software (MathWorks Inc., USA):

$$ {\text{EMC}} = {\text{MC}}_{0}  + {\text{MC}}_{{\text{f}}} \left( {1 - \exp \left( { - {\text{t/t}}_{{\text{f}}} } \right)} \right) + {\text{MC}}_{{\text{s}}} \left( {1 - \exp \left( { - {\text{t/t}}_{{\text{s}}} } \right)} \right) $$

where EMC stands for the equilibrium moisture content. MC_0_ is the initial moisture content. MC_f_ and MC_s_ are the moisture contents related to the fast and slow processes, respectively. The t_f_ and t_s_ are the times to reach equilibrium in the fast and slow processes, respectively.

### Measurement of bamboo cell dimensions

The samples were macerated following a previously reported method^23^. Samples of moso bamboo were split into small bamboo sticks with an approximate size of 10 mm (longitudinal) × 5 mm (radial) × 5 mm (tangential). The sticks were macerated in a solution containing equal parts of glacial acetic acid (99.5%) and hydrogen peroxide (30% solution) and were subsequently heated at 60 °C for 48 h. The maceration process ended when the color of the sticks turned white and could be easily broken into several parts. The segregated tissues were kept in a vial filled with ethanol (50%). Individual bamboo cells were then selected using fine-tipped tweezers under a dissecting microscope and placed on microscope slides to acquire temporary specimens. Next, the cells were observed by an optical microscope (Leica DM LB2), and the cell images were photographed using a digital camera (Leica DFC 300 FX). Image J software (National Institutes of Health, Bethesda, MD, USA) was used to measure the width and length of fiber and parenchyma cells and 100 cells of each type were measured.

### Fine structural observation

Stereomicroscope was used to observe the structure of the bamboo blocks, and Field Emission Scanning Electron Microscope (FE-SEM) (FE-SEM, JEOLJSM—6310F, JEOL, Tokyo, Japan) was used to observe the structure of bamboo slices.

## Results and discussion

### Dimensions of bamboo cells

**Figure 2:**
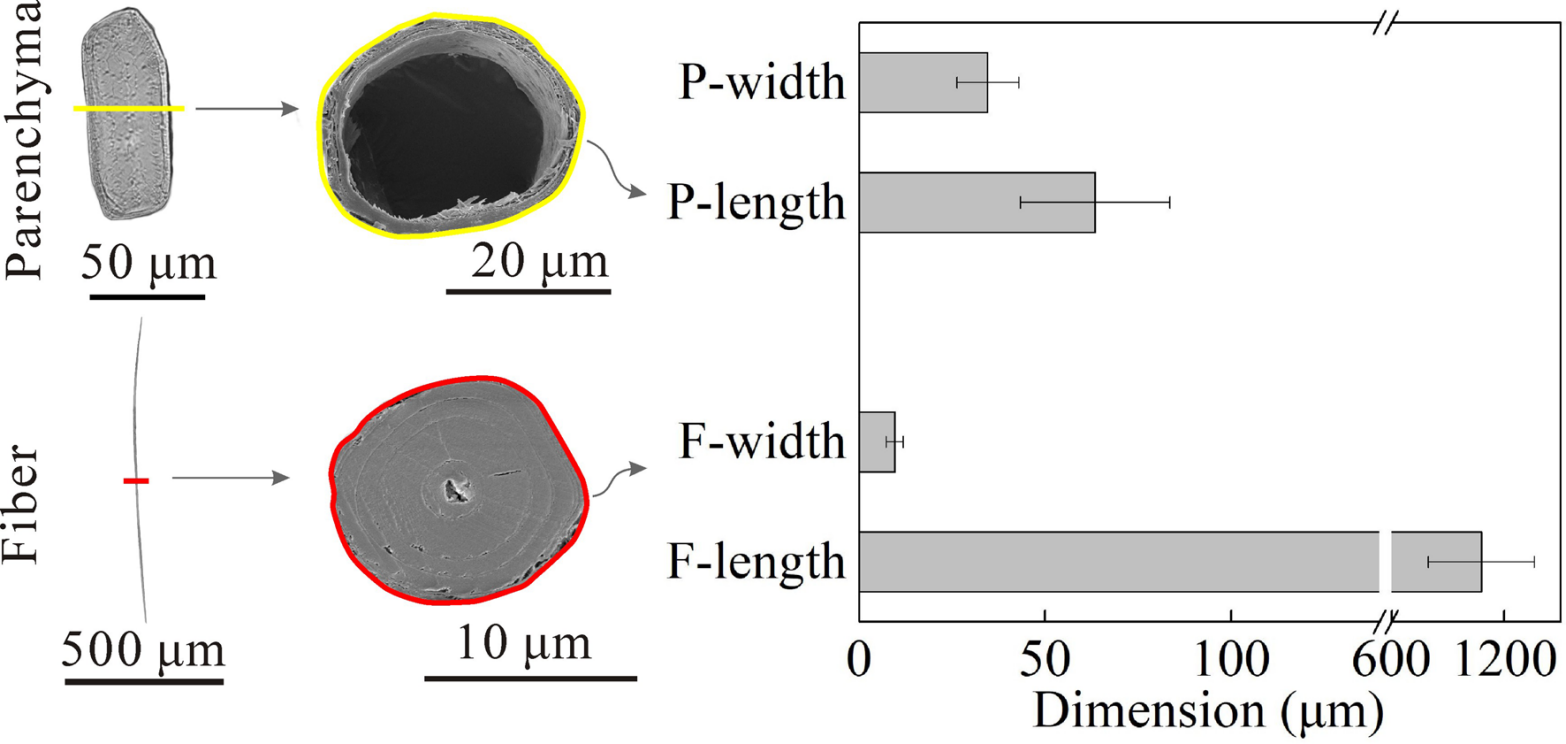
Front view and top view of a single parenchyma and fiber cells, and the diameters and lengths of parenchyma and fiber cells.

To investigate the sorption of bound-water in the cell wall substances, the dimensions of bamboo cells were determined first. The front view and top view of a single fiber and parenchyma cells are shown in Fig. 2. Fiber cells had slender outline and solid multilayer thick- walled structures with tiny lumens, while parenchyma cells had short and fragile multilayer thin- walled structures with big lumens. Their diameter and length are also shown in Fig. 2. The average dimension of a parenchyma cell was 63 × 35 μm (length × diameter), while 1077 × 10 μm for a fiber cell. The previous studies obtained close results^10^. There was no noticeable difference in fiber cells dimensions observed between the 30 μm and 60 μm bamboo slices. However, the morphologies for parenchyma cells in 30 μm slices were different from that in 60 μm slices, and its effects on sorption behavior is discussed in the following sections.

### Transverse/longitudinal bound-water sorption in cell wall substance

**Figure 3:**
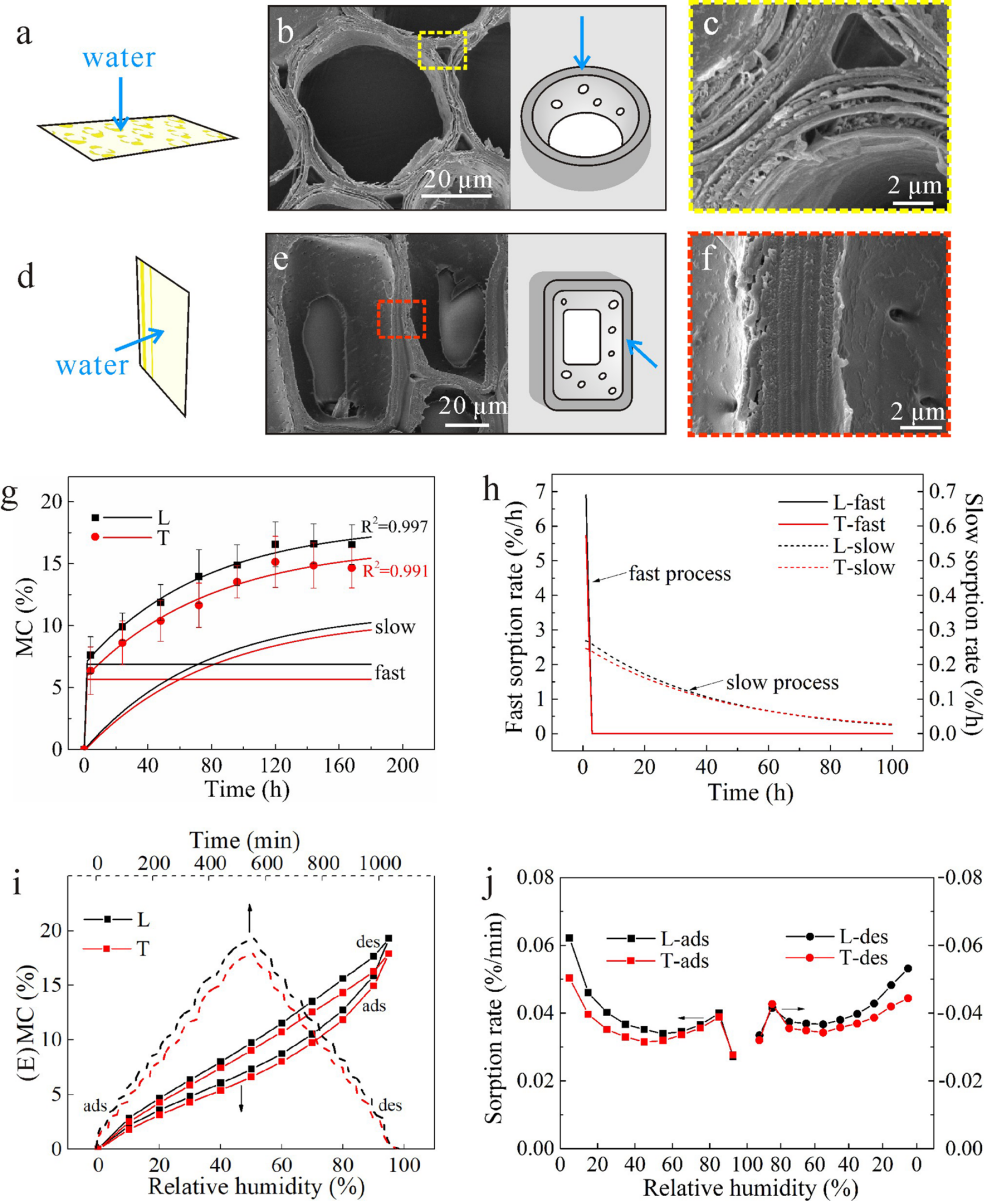
(**a**–**c**) Structural characterization of 30 μm specimen with longitudinal sorption direction: (**a**) graphical illustration of specimen, (**b**) SEM pictures of parenchyma cells, (**c**) SEM pictures of parenchyma cells walls. (**d**–**f**) Structural characterization of 30 μm specimen with transverse sorption direction: (**d**) graphical illustration of specimen, (**e**) SEM pictures of parenchyma cells, (**f**) SEM pictures of parenchyma cells walls. (**g**–**j**) Sorption behaviors of 30 μm specimens: (**g**) experimental data (dots) of MC over time by the saturated salt solution method, the PEK fitted curves (lines), and slow and fast processes of PEK model, (**h**) Sorption rates of fast and slow processes, (**i**) The DVS data of MC change during whole sorption process (MC change with time from 0 to 1200 min, and EMC change with RH from 0 to 95%), (**j**) sorptions rates during RH from 0 to 95%.

When the slice thickness was 30 μm (Fig. 3a,d), there was only a segment of the parenchyma cells left, and they appeared like hollow drums without ends, regardless of the L or T directions (Fig. 3b,e). Therefore, the sorption behavior was considered only for transport in the cell wall and not in the cell lumen, and the effect of pits has been ignored because the lumen was exposed to water vapor and the moisture content (MC) change was mainly due to water sorption by the cell walls. Thus, the results of water sorption in 30 μm slice of bamboo could be regarded as the bound-water sorption rate of the cell wall substances.

The dots in Fig. 3g are the MC change with time in two directions as measured using the saturated salt solution method. MC increased rapidly during the early stage of sorption and reached to a steady state after 120 h. The difference in MC between T and L directions almost occurred at the onset and remained relatively constant throughout the sorption process. Even with some overlaps between the error bars, the average sorption trends appeared to be noticeably different between the T and L specimens. In a previous study, Hartley et al.^24^ explained that the moisture increment was dominated by sorption sites at the initial sorption period. In this regard, more sorption sites were readily available for water interaction in the L specimens than those in the T specimens. The parenchyma cells walls in two specimens are shown in Fig. 3c,f. The cross-cut cell wall in L specimen was looser than longitudinally cut cell wall in the T specimen. This might result in the exposure of greater number of hydroxy groups to water in the L specimens. L specimens always showed higher MC values during the entire sorption process. In Fig. 3i, the dotted line is the MC change with time while the solid line is the EMC change with RH. The difference between the L and T specimens mainly occurred in the earlier stage (0–400 min), which corresponded to the RH from 0 to 40%. At low RH, water sorption was mainly attributed to hydroxy groups in the cell wall, forming monolayer of water^17,24,25^. A higher EMC indicated that the L specimens had more sorption sites than the T specimens.

The water vapor sorption kinetics was analyzed by the PEK model, and the fitted curves to the experimental data are shown in Fig. 3g. Both the fits had R^2^ values greater than 0.99. PEK curves are divided into fast and slow processes (Fig. 3g). The fast MC increased rapidly initially and remained constant once it reached to 5–7% in 3 h. On the contrary, the slow MC increased during the whole period and increased approximately 10% MC, which suggested that the EMC was dominated by the slow process. Xie et al.^22^ obtained similar results in wood materials. Besides, L specimens always had a larger MC than T specimens, regardless of fast or slow processes. The fast process is governed by a physical sorption phenomenon (adsorption onto readily accessible OH sites), whereas the slow process is linked to relaxation processes associated with cell wall expansion or contraction^19,20,22,26^. The L specimens had a looser cell wall layer (Fig. 3c,f), which provided more exposure for interaction with water molecules, thus greater cell swelling.

Sorption rates of fast and slow processes were obtained by taking the derivative of PEK curves, and the results are shown in Fig. 3h. The fast sorption rate was highest initially, reaching 7%/h for L specimens. Then, it decreased quickly and became almost 0 after 3 h. Slow sorption rates were far lower than fast sorption rates initially with the highest value being approximately 0.3%/h. Then, it decreased slowly over time. For both fast and slow sorption rates, L specimens were higher than T specimens during the initial period. For the fast process, L and T specimens had similar sorption rates after 3 h, while it was after 50 h for the slow process. The sorption rates during RH from 0 to 95% were obtained by dividing the increment or decrement of EMC by time (Fig. 3i), and the results are shown in Fig. 3j. Sorption rate of L specimens was always higher than that of T specimens, approximately 1.2 times at the low RH (10%), which was similar to wood^27,28^. In the adsorption process, the sorption rate of T specimen was slightly higher than that of L specimen at 95% RH. This might be a random error due to the large DVS setting of the weight change (0.01%). The difference between L and T specimens is believed to be attributed again to the tightness of cell wall layers (Fig. 3c,f) and thus availability of hydroxyl groups to water molecules during the sorption process.

### Effect of pits

**Figure 4:**
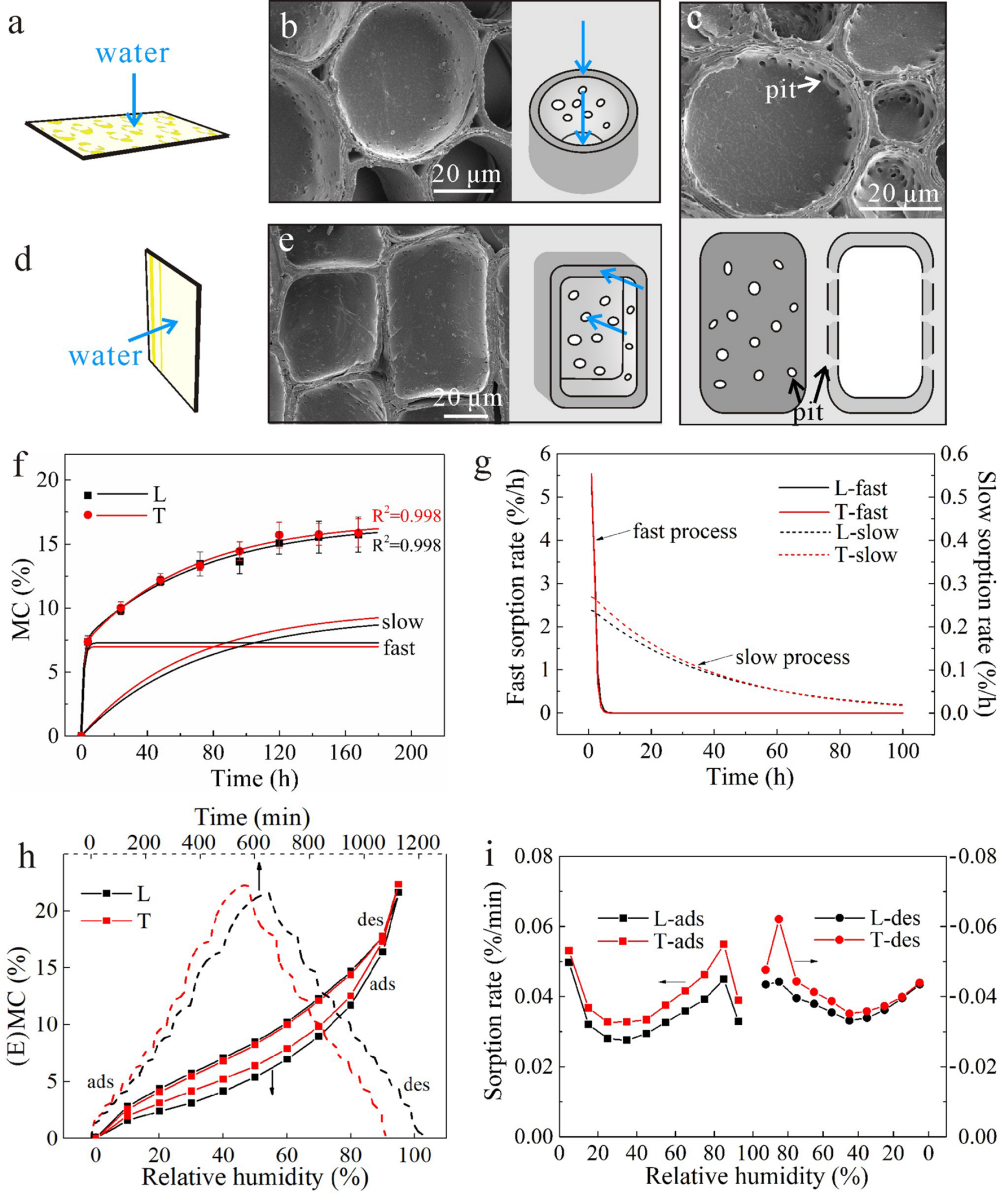
(**a**, **b**) Structural characterization of 60 μm specimen with longitudinal sorption direction: (**a**) graphical illustration of specimen, (**b**) SEM pictures of parenchyma cells. (**c**) The distribution of pits in parenchyma cells. (**d**, **e**) Structural characterization of 60 μm specimen with transverse sorption direction: (**d**) graphical illustration of specimen, (**e**) SEM pictures of parenchyma cells. (**f**–**i**) Sorption behaviors of 60 μm specimens: (**f**) Experimental data (dots) of MC over time by the saturated salt solution method, the PEK fitted curves (lines), and slow and fast processes of PEK model, (**g**) Sorption rates of fast and slow processes, (**h**) The DVS data of MC during whole sorption process (MC change with time from 0 to 1300 min, and EMC change with RH from 0 to 95%), (**i**) Sorptions rates during RH from 0 to 95%.

Compared with 30 μm specimens, specimens with 60 μm thickness (Fig. 4a,d) contained half the structure of parenchyma cells (Fig. 4b,e), especially in the T direction (Fig. 4e). In this case, when water transporting along the thicknesses of the thicker specimens, water molecules needed to go across the cell walls (Fig. 4b,e) where the effect of pits could not be ignored, but the effect of water diffusion in the cell lumen was not considered as almost no entire cell was observed. The pits in parenchyma cells were only distributed in the lateral walls, not in the end walls (Fig. 4c)^29^ and thus L specimens contained fewer pits than in T specimens along diffusion direction.

The MC with error bars for 60 μm specimens with two sorption directions changed with time is shown as dots in Fig. 4f. Due to the effect of pits, the MCs of T and L specimens were almost the same initially, but T specimens showed slightly higher MCs over time. This result was different from the result of 30 μm specimens where L specimens had a higher MC when only the bound-water sorption of cell wall substance was considered. The difference between the two thickness specimens is believed to be caused by the effect of pits. Besides, in the RH range of 0–95%, T specimens had a higher EMC (Fig. 4h). This could be attributed to the presence of more pits in the T specimen, causing bigger contact areas and spaces for water molecules, thus resulting in higher EMC.

The fast and slow processes, according to the PEK model, are also shown in Fig. 4f, and the sorption rates of fast and slow processes are shown in Fig. 4g. In the fast process, there was no noticeable difference between T and L specimens; while in the slow process, the MC and sorption rate of T specimens were higher than that of L specimens. The sorption rates of T and L specimens in the RH range of 0–95% are shown in Fig. 4i, with the former being always higher than the latter, especially at high RH. All these results indicated that the existence of pits created more sorption spaces for water^17^. Siau^27,28^ did not consider pits as an important factor for water sorption in wood, below the fiber saturation point. However, in bamboo, since there was no transverse transport tissue, the effect of pits was essential and could not be overlooked.

### Transverse/longitudinal water sorption in bulk bamboo

**Figure 5:**
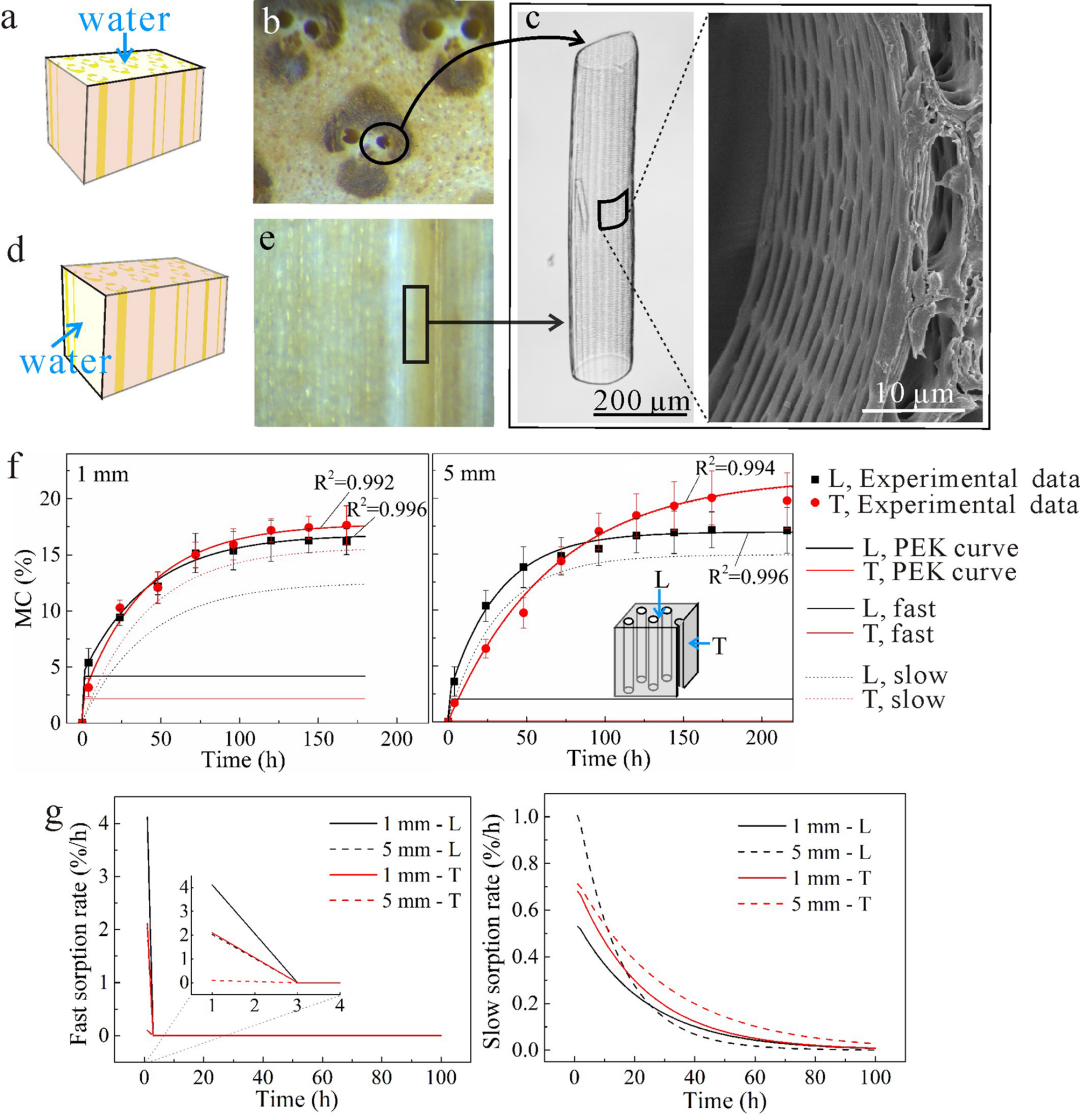
(**a**, **b**) Structural characterization of 5 mm specimen with longitudinal sorption direction: (**a**) Graphical illustration of specimen, (**b**) Optical micrograph pictures of sorption surface. (**c**) The front view of a single vessel cell and top view of vessel cell in specimens. (**d**, **e**) Structural characterization of 5 mm specimen with transverse sorption direction: (**d**) Graphical illustration of specimen, (**e**) Optical micrograph pictures of parenchyma cells. (**f**, **g**) Sorption behaviors of bamboo block (1 and 5 mm) with different sorption directions (T and L): (**f**) experimental data (dots) of MC over time by the saturated salt solution method, PEK fitted curves (lines),and slow and fast processes of PEK model, (**g**) sorption rates of fast (left) and slow (right) processes.

When the thickness of the specimen was over 1 mm, most of the cells were intact, and thus it was necessary to include the sorption of bound-water in cell wall substance, the effect of pits, and the water vapor diffusion in the cell lumen to account for the total sorption behavior. The MC values with error bars for bamboo blocks with two thicknesses (1 and 5 mm) and two sorption directions are shown as dots in Fig. 5f. During the initial sorption period, MC of L specimens was higher than that of T specimens, while contradictory results were obtained after a certain period of time, i.e., 24 h for 1 mm and 100 h for 5 mm. These were caused by the vessel cells. Vessel cells had big lumens and thin cell walls with many pits and most of the vessel cells had open ends (Fig. 5c). The water–vapor diffusion coefficient in cell lumen was much higher than the bound-water diffusion coefficient in cell wall^27^. For the L specimens (Fig. 5b), water could access the inside of the vessel cell lumen through its end surfaces, and then into bamboo cell wall through the pits in the lumen cell wall (Fig. 5c). However, for the T specimen (Fig. 5e), water could only access the inside of the bamboo by gradual diffusion along the lateral cell wall. Thus, MC of L specimens was higher initially. Nevertheless, at the end of the experiment, the MC of L specimens was lower (Fig. 5f). This was because the lumen of vessel cells in L specimens was open to the air. When the end surfaces of vessel cell were open (Fig. 5a,b), the weight of water vapor in cell lumen was ignored. On the contrary, for the T specimens, both the end surfaces were covered (Fig. 5d,e) and the weight of the water vapor in the cell lumen was considered. Thus, the MC of L specimens was lower. The difference between the L and T specimens was bigger when the specimen is thicker (Fig. 5f) regardless of the time. This indicated that the effect of vessel cells increased as the thickness of the specimens increased since the thicker specimens contained more vessel cells. Note that minor experimental errors could occur due to larger relative moisture loss in the thinner specimens than thicker specimens during weighing process outside the desiccators.

PEK model fitted to the experimental data are shown as the lines in Fig. 5f, with R^2^ values above 0.99. The fast and slow processes of specimens are also shown in Fig. 5f. The fast MC increased rapidly initially, and then was constant once MC reached to 3–5%. In contrast, slow MC increased during the whole period and reached 15–20% MC, which suggested that the total EMC was dominated by the slow process. Compared with 1 mm samples, the 5 mm samples had a smaller MC_f_ but a bigger MC_s_ (Fig. 5f). This indicated that the slow process became more essential as the thickness of the sample increased. The fast and slow sorption rates of 1 mm and 5 mm specimens are shown in Fig. 5g and demonstrated a similar trend to the 30 and 60 μm specimens. Sorption rates of L specimens were higher than those of T specimens in the fast process while was lower in slow processes, which were also caused by the vessel cells.

### Water transport at various hierarchical scales

**Figure 6:**
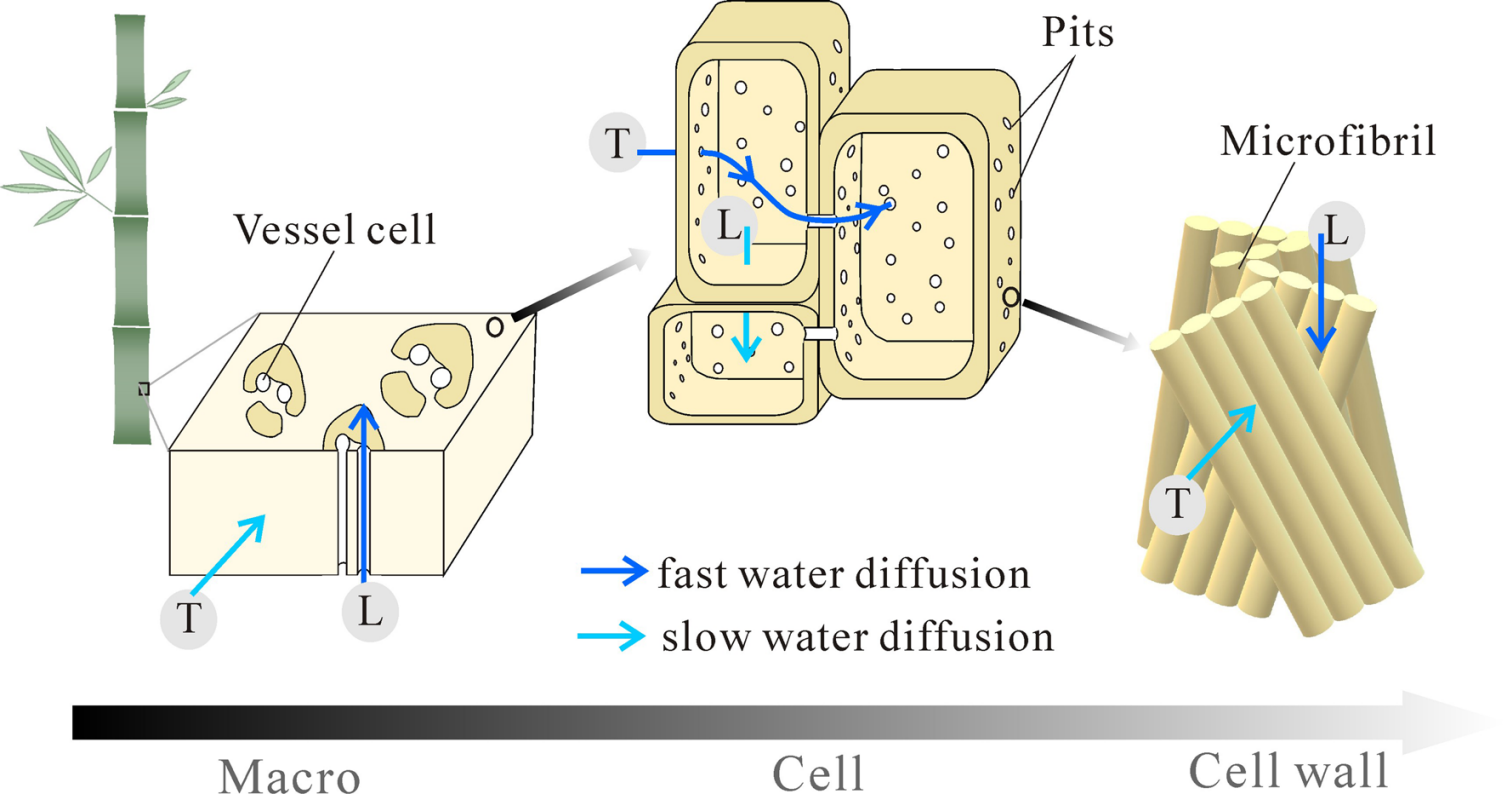
Schematic illustration of multi-scale water vapor diffusion paths in bamboo.

At the cell wall scale, when water transported in the cell wall matrix, the sorption rate in L direction was higher than that in T direction. However, at slightly greater scale where the effect of pits was considered, the reverse trend could emerge where the sorption rate in T direction might be higher than L. Then again, at the macro-scale, the sorption rate in L direction was higher than that in T direction. These different sorption behaviors at various scales probably stemmed from the different biological needs for water transport in bamboo as a plant. Although the water transport in a living bamboo is liquid, the transport network is believed to be also conducive for vapor diffusion as well. As in other plants, the formation of vessel cells allows water to transport in bamboo upwards from the roots to the leaves to participate in the biochemical functions (Fig. 6). Thus, the sorption rate in L direction was higher than that of T direction. At the cellular scale, water transports along the transverse direction between the parenchyma cells through pits. The pits enhance the localized sorption rate across the cell walls or along transverse direction (Fig. 6).

By analyzing the sorption rates in specimens of characteristically different dimensions and orientations, this work allows the global and local water transport behavior in bamboo to be linked to its cellular structure features at three scale levels: cell walls, pits cross the cell wall and cell lumens. The novelty of this approach also lies in the fact that it allows for elucidation of contributions to the liquid transport in bamboo as a plant or material by its major constituents, which are by order vessels, parenchyma cell via pits and fibers.

The structure-induced variability of water sorption is a major problem in bamboo drying, causing uneven drying rate, differential shrinkages and drying stresses. This will result in dimensional instability (fiber twisting, warping or cupping) during drying process^30–32^. On the other hand, water sorption rate is also directly related to the ability to treat bamboo, e.g., mold resistant treatment using borate solution, both in terms of the treatment time and uniformity^33^. In addition, the water sorption responses at different structure levels will have an impact on the multiscale mechanical properties of bamboo^34–37^, through the effect of moisture content or EMC^38,39^ and dynamic moisture diffusivity.

## Conclusions

Water sorption process in bamboo was a complex transport response occurring in multiple scales. At the macro scale, the transport of moisture took place mainly along the longitudinal direction; whereas at the cellular scale, the moisture needed to transport in the transverse direction. Based on this study, the water vapor sorption behavior of bamboo at various hierarchical scales can be summarized as follows.

1. At the cell wall level (sample thickness 30 μm), the bound water diffused in cellular solids, and the sorption rate and EMC of cell walls were greater in the longitudinal specimens than in the transverse specimens. This was due to the looser packing of cell wall constituents and the more exposed sorption sites for interaction with water.
2. As the specimen thickness increased to 60 μm, the inter-cellular sorption involved more transport in the space of pits. Since the vapor diffusivity in the void space is much greater than bound water diffusivity within the cell walls^27^, the pits play a more dominant role in governing the sorption rate. The pits in parenchyma cells were only distributed in the lateral walls, and thus the pits increased moisture sorption in the transverse specimens.
3. At the macro scale with thick (1 and 5 mm) specimens, the space of lumens of vessel cells formed the main pathways in transporting moisture along the longitudinal direction of bamboo block. Thus, the sorption rate and EMC were greater in the longitudinal specimens than the transverse specimens. The effect of lumens of vessel cells on sorption rates between longitudinal and transverse directions increased with the thickness of specimen.
